# Old Friends.—Regularity of Life

**Published:** 1855-01

**Authors:** 


### OLD FRIENDS.—REGULARITY OF LIFE.

Forty years ago this present month of October, two young merchants of this city, who had been school-fellows together, met on a bright autumnal afternoon, after the business of the day was over, in front of the old Tontine Coffee House, and as they walked up Wall street together, one said to the other, "Come and dine with me to-day. Monday always seems a dull day, and my wife and I like to have some one drop in socially and dine or take tea with us." The invitation was accepted, and after dinner, as they sat over their wine (limited to a couple of glasses each,) and talked of their school-days and of their pleasant intercourse in the years that succeeded, they agreed to dine with each other on alternate Mondays, unless prevented by sickness or other causes, so long as they should live. Time passed on; sons and daughters were born to them, grew up, married and settled in life; grand children, too, have not been wanting. New York has grown from one of the plainest of little cities to be one of the great cities of the world; her commerce has increased beyond the wildest dream of imagination in former days; hotels and warehouses line her streets, steamers and other shipping her wharves, and private dwellings of more than princely elegance her up-town squares and avenues, all on a scale of such magnificence, that he who had prophesied of such things, would once have been thought an enthusiastic dreamer. Our whole country and indeed the world, has changed in the same ratio, until the mind is lost in conjecturing what two score more of years will produce. Eleven different Presidents have governed our own nation, and the nations of the world have changed their rulers from revolutions and other causes, again and again. Such are the changes time has made, and still these two old friends hale and healthy yet, may be seen on every Monday afternoon, going up the street together to fulfil the mutual promise they made to each other in early manhood. They set a high standard, at the commencement of their mercantile life, of which an honorable, upright merchant should be, and have never departed from it; and if they have not been among the wealthiest of the merchant princes of the Empire City, they have had enough,—and have spared not a little when the demands of public and private charities have been presented to them.—May they long live to enjoy life as well as at this present period, and set the same good example to younger men to follow in their footsteps.—*N. Y. Express.*

